# Virological failure and treatment switch after ART initiation among people living with HIV with and without routine viral load monitoring in Asia

**DOI:** 10.1002/jia2.25989

**Published:** 2022-08-26

**Authors:** Sirinya Teeraananchai, Matthew Law, David Boettiger, Nicole De La Mata, Nikhil Gupte, Yun‐ting Lawrence Chan, Thach Ngoc Pham, Romanee Chaiwarith, Penh Sun Ly, Yu‐Jiun Chan, Sasisopin Kiertiburanakul, Suwimon Khusuwan, Fujie Zhang, Evy Yunihastuti, Nagalingeswaran Kumarasamy, Sanjay Pujari, Iskandar Azwa, I Ketut Agus Somia, Junko Tanuma, Rossana Ditangco, Jun Yong Choi, Oon Tek Ng, Cuong Duy Do, Yasmin Gani, Jeremy Ross, Awachana Jiamsakul, the TREAT Asia HIV Observational Database (TAHOD) of IeDEA Asia‐Pacific

**Affiliations:** ^1^ Department of Statistics Faculty of Science Kasetsart University Bangkok Thailand; ^2^ HIV‐NAT, Thai Red Cross AIDS Research Centre Bangkok Thailand; ^3^ The Kirby Institute UNSW Sydney New South Wales Australia; ^4^ Sydney School of Public Health The University of Sydney Sydney New South Wales Australia; ^5^ BJ Government Medical College and Sassoon General Hospital Pune India; ^6^ Queen Elizabeth Hospital Kowloon Hong Kong SAR; ^7^ National Hospital for Tropical Diseases Hanoi Vietnam; ^8^ Chiang Mai University ‐ Research Institute for Health Sciences Chiang Mai Thailand; ^9^ Division of Infectious Diseases and Tropical Medicine Department of Medicine Faculty of Medicine Chiang Mai University Chiang Mai Thailand; ^10^ National Center for HIV/AIDS, Dermatology & STDs Phnom Penh Cambodia; ^11^ Taipei Veterans General Hospital Taipei Taiwan; ^12^ Faculty of Medicine, Ramathibodi Hospital Mahidol University Bangkok Thailand; ^13^ Chiangrai Prachanukroh Hospital Chiang Rai Thailand; ^14^ Beijing Ditan Hospital Capital Medical University Beijing China; ^15^ Faculty of Medicine, Universitas Indonesia ‐ Dr. Cipto Mangunkusumo General Hospital Jakarta Indonesia; ^16^ Chennai Antiviral Research and Treatment Clinical Research Site (CART CRS) VHS‐Infectious Diseases Medical Centre, VHS Chennai India; ^17^ Institute of Infectious Diseases Pune India; ^18^ University Malaya Medical Centre Kuala Lumpur Malaysia; ^19^ Faculty of Medicine, Udayana University & Sanglah Hospital Bali Indonesia; ^20^ National Center for Global Health and Medicine Tokyo Japan; ^21^ Research Institute for Tropical Medicine Muntinlupa City Philippines; ^22^ Division of Infectious Diseases Department of Internal Medicine Yonsei University College of Medicine Seoul South Korea; ^23^ Tan Tock Seng Hospital Singapore; ^24^ Bach Mai Hospital Hanoi Vietnam; ^25^ Hospital Sungai Buloh Sungai Buloh Malaysia; ^26^ TREAT Asia, amfAR ‐ The Foundation for AIDS Research Bangkok Thailand

**Keywords:** HIV, routine viral load testing, Asia, cohort studies, antiretroviral therapy, virological failure

## Abstract

**Introduction:**

Viral load (VL) testing is still challenging to monitor treatment responses of antiretroviral therapy (ART) for HIV treatment programme in Asia. We assessed the association between routine VL testing and virological failure (VF) and determine factors associated with switching to second‐line regimen.

**Methods:**

Among 21 sites from the TREAT Asia HIV Observational Database (TAHOD), people living with HIV (PLHIV) aged ≥18 years initiating ART from 2003 to 2021 were included. We calculated the average number of VL tests per patient per year between the date of ART initiation and the most recent visit. If the median average number of VL tests was ≥ 0.80 per patient per year, the site was classified as a routine VL site. A site with a median < 0.80 was classified into the non‐routine VL sites. VF was defined as VL ≥1000 copies/ml during first‐line therapy. Factors associated with VF were analysed using generalized estimating equations with Poisson distribution.

**Results:**

Of 6277 PLHIV starting ART after 2003, 3030 (48%) were from 11 routine VL testing sites and 3247 (52%) were from 10 non‐routine VL testing sites. The median follow‐up was 9 years (IQR 5–13). The median age was 35 (30–42) years; 68% were male and 5729 (91%) started non‐nucleoside reverse‐transcriptase inhibitor‐based regimen. The median pre‐ART CD4 count in PLHIV from routine VL sites was lower compared to non‐routine VL sites (144 vs. 156 cells/mm^3^, *p* <0.001). Overall, 1021 subsequent VF at a rate of 2.15 (95% CI 2.02–2.29) per 100 person‐years (PY). VF was more frequent at non‐routine VL sites (adjusted incidence rate ratio 2.85 [95% CI 2.27–3.59]) compared to routine VL sites. Other factors associated with an increased rate of VF were age <50 years and CD4 count <350 cells/mm^3^. A total of 817 (13%) patients switched to second‐line regimen at a rate of 1.44 (95% CI 1.35–1.54) per 100 PY. PLHIV at routine VL monitoring sites were at higher risk of switching than those at non‐routine VL sites (adjusted sub‐hazard ratio 1.78 95% CI [1.17–2.71]).

**Conclusions:**

PLHIV from non‐routine VL sites had a higher incidence of persistent VF and a low switching regimen rate, reflecting possible under‐utilized VL testing.

## 1 INTRODUCTION

The scale‐up of antiretroviral therapy (ART) had a substantial positive impact on the health and quality of life of people living with HIV (PLHIV) and significantly reduced the incidence of HIV infection. There were almost 38 million PLHIV globally at the end of 2020, and 74% of adults living with HIV had access to ART [1]. Increasing treatment cascades towards 90‐90‐90 UNAIDS targets are indicators of the effective performance of long‐term HIV treatment programmes. Achieving viral load (VL) suppression is key to the success of ART at the individual level. Routine VL testing is recommended for treatment monitoring, and to assess whether treatment switches are necessary [2]. According to the World Health Organization (WHO) recommendations, routine VL testing should be conducted 6 and 12 months after ART initiation and every 12 months thereafter [3]. Previous studies indicate that VL tests can detect treatment failure earlier and more accurately than CD4 testing or clinical monitoring [4, 5, 6]. However, in resource‐limited settings, routine VL testing may not be readily available and switches to a second‐line regimen may occur immediately without evidence of virological failure (VF). However, previous studies report that PLHIV from sites with routine VL testing switched to second‐line ART after virologic failure was confirmed [6, 7, 8, 9]. Studies from Asia indicate that routing VL testing can improve clinical outcomes [6, 9, 10]. Most studies were conducted prior to the WHO recommendations of including dolutegravir (DTG) in first‐line combinations as well as in second‐line for those who are failing non‐DTG‐based regimens [11]. Studies on the effects of routine VL testing on treatment switches have not been conducted extensively in Asia, where the majority of patients would most likely be switching from a non‐nucleoside reverse‐transcriptase inhibitor (NNRTI)‐based combinations.

The TREAT Asia HIV Observational Database (TAHOD) of IeDEA Asia‐Pacific (International Epidemiology Databases to Evaluate AIDS) is a longitudinal observational cohort study, which monitors long‐term treatment outcomes in PLHIV in Asia. VL monitoring varies across TAHOD sites. In this study, we aimed to assess the association between routine VL testing (routine VL) and VF during first‐line therapy and investigate factors associated with switching to a second‐line regimen.

## 2 METHODS

### 2.1 Study population

As TAHOD is an adult HIV observational cohort, our analysis included PLHIV aged ≥18 years at ART initiation, from January 2003 to September 2021. There were 21 sites from Cambodia, China, Hong Kong, India, Indonesia, Japan, Malaysia, Philippines, Singapore, South Korea, Taiwan, Thailand and Vietnam. The TAHOD cohort contributes to the IeDEA global consortium and has been described previously [12, 13, 14]. TAHOD sites are predominantly major HIV referral centres with data collected during routine care [12, 13]. Patients were included if they had started ART with at least three drugs, including an NNRTI or protease inhibitor (PI), and 2–3 nucleoside reverse‐transcriptase inhibitors (NRTIs); had at least one VL test in the first year after ART initiation (to allow sufficient follow‐up time after treatment); and at least one VL during follow‐up. We included all follow‐ups based on VL tests during follow‐up of first‐line therapy as a longitudinal cohort study. Ethics approval was obtained at the sites, TREAT Asia/amfAR (coordinating centre), and the Kirby Institute (data management and statistical analysis centre). A consent waiver was obtained for this study.

### 2.2 Definition and outcomes

We classified sites according to whether they performed routine VL testing, that is routine VL versus non‐routine VL. We defined routine VL by calculating the average number of VL tests for each patient between ART initiation and the last visit date. If the median of the average number of VL tests was above 0.80 per patient per year, the site was classified as routine VL site. If the median was less than 0.80, the site was classified as a non‐routine VL site. We utilized 0.80 as the cut of as the WHO's recommendation for VL testing was done once a year. As there would have been some variability in appointment scheduling and attendance, we utilized 0.80 as the cut‐off to allow some flexibility associated with the WHO's guidelines [2, 14, 15]. In our first analysis, we defined first VF as the first occurrence of VL ≥1000 copies/ml after 6 months on first‐line ART. Subsequent VF was defined as any VL ≥1000 copies/ml occurring at any time after the first VF while on first‐line ART. The follow‐up time of first‐line therapy was censored on the date of the last visit or the date of switching to second‐line ART for those who have switched. In our second analysis, we defined a switch to second‐line ART as a change in a major drug class from NNRTI to PI or vice‐versa for at least 6 months, due to treatment failure defined as having at least one VF and used the date of VF closest to the date of switching. PLHIV who did not have VF was confirmed from medical recordings, including immunological and clinical failure for switching to second‐line ART. Lost to follow‐up (LTFU) was defined as not having a clinic visit in the previous 12 months. The baseline time point was defined as the date of ART initiation.

### 2.3 Statistical analysis

Baseline characteristics, including demographics, age, income country level, HIV exposure, hepatitis C co‐infection, prior AIDS‐defining illness, year of ART initiation, first‐line ART regimen and pre‐ART CD4 count level, were summarized using descriptive statistics for routine VL and non‐routine VL sites. The comparison of characteristics between routine VL and non‐routine VL sites was performed using Pearson's Chi‐square or Fisher's exact test, as appropriate, for categorical data and Wilcoxon rank sum test for continuous data. The outcomes rate was calculated by dividing the total number of outcomes by the total number of person‐years (PY) of follow‐up and expressed as a rate per 100 PY. The associations between routine VL status including with other predictors and VF were presented as incidence rate ratios (IRR) by using generalized estimating equation (GEE) with log link function (Poisson distribution) and exchangeable correlation structure. We used Fine and Gray's competing risk regression [16] to assess the associations between predictors and switching to second‐line ART. Death and LTFU were defined as competing events. Covariates assessed in both GEE and competing risks models included gender, VL routine status, income country level, current age, HIV exposure, HCV status, prior AIDS‐defining illness, year of ART initiation, first‐line ART regimen and current CD4 count level.

Variables with *p* <0.10 in the univariate analysis were considered for inclusion in multivariate models. Statistical significance was identified using a two‐sided *p* value less than 0.05. Statistical analysis was performed with SAS version 9.4 (SAS Institute Inc, Cary, NC) and with Stata version 16 (Stata Corp, College Station, TX).

## 3 RESULTS

### 3.1 Patient characteristics

**Figure 1:**
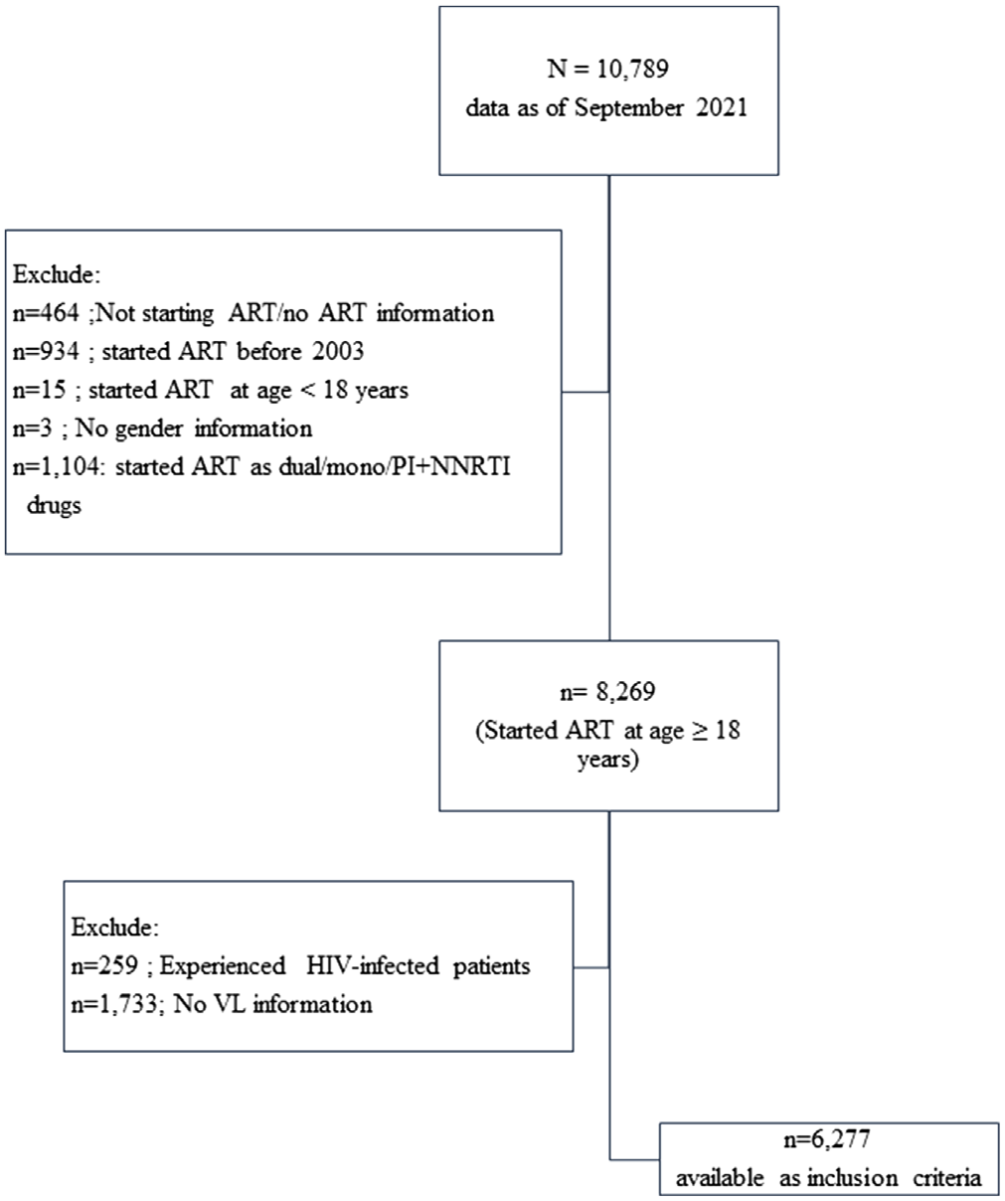
Study profile. Abbreviations: ART, antiretroviral therapy; NNRTI, non‐nucleoside reverse transcriptase inhibitor; PI, protease inhibitor; VL, viral load.

A total of 10,789 patients were included. Of these, 4512 patients were excluded for the following reasons: (1) not having yet initiated ART (*n* = 464, 4%), (2) starting ART with only mono/dual or combination of NNRTI and PI drugs (*n* = 1104, 10%), (3) initiating ART before 2003 (*n* = 934, 9%), (4) age <18 years at ART initiation (*n* = 15, 0.5%), (5) no sex information (*n* = 3, 0.5%), (6) assumed to be treatment experienced due to VL <50 copies/ml at baseline (*n* = 259, 2%) and (7) no VL measurement after ART initiation (*n* = 1733, 16%) (Figure 1).

A total of 6277 patients were eligible for the study. There were 3030 (48%) patients from 11 routine VL testing sites which consisted of five sites from high‐income countries and six sites from upper‐middle‐income countries, and 3247 (52%) patients from 10 non‐routine VL sites (eight in lower‐middle‐income countries and two in upper‐middle‐income countries). The majority were male (65% in non‐routine VL, 71% in routine VL, *p* <0.001), and the median age at ART initiation was 35 (interquartile range, IQR 30–42) years. We found that 91% started with NNRTI‐based ART (99% (58% nevirapine [NVP], 41% efavirenz [EFV]) in lower middle‐income; 95% [47% NVP, 48% EFV] in upper middle‐income; and 60% [11% NVP, 49% EFV] in high‐income countries) and 9% started with PI‐based ART (1% (0.3% lopinavir [LPV], 0.7% atazanavir [ATV]) in lower middle‐income; 5% [3% LPV, 2% ATV] in upper middle‐income; and 40% [24% LPV, 16% ATV] in high‐income countries). Most patients had a heterosexual mode of HIV exposure. Thirty‐six percent had a prior AIDS diagnosis. The median pre‐ART CD4 count in patients from routine VL sites was higher compared to non‐routine VL sites (150 vs. 131 cells/mm^3^, *p* <0.001). Forty percent had pre‐ART CD4 < 200 cells/mm^3^ (42% in non‐routine VL vs. 38% in routine VL). We found that 42% had pre‐ART CD4 < 200 cells/mm^3^ with advanced HIV disease stage. There was no significant difference in the proportion of pre‐ART VL ≥1000 copies/ml between non‐routine VL and routine VL sites (Table 1).

**Table 1:** Characteristics of HIV‐infected patient at ART initiation

Characteristics	Non‐routine VL	Routine VL	Total	*p*
*N*	3247 (52%)	3030 (48%)	6277	
Site	10	11	21	
Income country level, *n* (%)				
High income	2579 (79)	0 (0)	2579 (41)	<0.001
Upper to middle income	668 (21)	2057 (68)	2725 (43)	
Lower middle income	0 (0)	973 (32)	973 (16)	
Male, *n* (%)	2096 (65)	2164 (71)	4260 (68)	<0.001
Median (IQR) age, years	35 (30–41)	36 (30–43)	35 (30–42)	<0.001
18 to <25, *n* (%)	227 (7)	269 (9)	496 (8)	<0.001
25 to <35, *n* (%)	1403 (43)	1097 (36)	2500 (40)	
35 to <50, *n* (%)	1345 (41)	1342 (44)	2687 (43)	
≥ 50, *n* (%)	272 (8)	322 (11)	594 (9)	
HIV exposure, *n* (%)				
Homosexual	362 (11)	996 (33)	1358 (22)	<0.001
Heterosexual	2333 (72)	1757 (58)	4090 (65)	
IDU	94 (3)	42 (1)	136 (2)	
Other	458 (14)	235 (8)	693 (11)	
Prior AIDS diagnosis, *n* (%)				
Yes	926 (29)	1113 (37)	2039 (32)	<0.001
No	384 (12)	202 (7)	586 (9)	
Unknown	1937 (60)	1715 (57)	3652 (58)	
Hepatitis B surface antigen status, *n* (%)				
Negative	1379 (42)	1502 (50)	2881 (46)	<0.001
Positive	148 (5)	183 (6)	331 (5)	
Unknown	1720 (53)	1345 (44)	3065 (49)	
Hepatitis C antibody status, *n* (%)				
Negative	1051 (32)	1461 (48)	2512 (40)	<0.001
Positive	299 (9)	89 (3)	388 (6)	
Unknown	1897 (58)	1480 (49)	3377 (54)	
First major regimen, *n* (%)				<0.001
NNRTI‐based ART	3147 (97)	2582 (85)	5729 (91)	
PI‐based ART	100 (3)	448 (15)	548 (9)	
Year of ART initiation, *n* (%)				
2003–2007	1075 (33)	1190 (39)	2265 (36)	<0.001
2008–2013	1646 (51)	1562 (52)	3208 (51)	
2014–2017	323 (10)	193 (6)	516 (8)	
2017–2021	203 (6)	85 (3)	288 (5)	
Pre‐ART CD4 count, *n* (%)	2230 (69)	1811 (60)	4041 (64)	
Median (IQR) CD4 count, cells/mm^3^	156 (56–264)	144 (42–246)	151 (50–253)	0.001
<200	1360 (42)	1165 (38)	2525 (40)	<0.001
200–350	595 (18)	480 (16)	1075 (17)	
≥350	275 (8)	166 (5)	441 (7)	
Unknown	1017 (31)	1219 (40)	2236 (36)	
HIV viral load available	848 (26)	1970 (65)	2818 (45)	
Median (IQR) log_10_ VL, copies/ml	5.04 (4.35–5.5)	4.96 (4.46–5.4)	4.98 (4.43–5.44)	0.13
HIV viral load ≥1000 copies/ml	799 (94)	1879 (95)	2678 (95)	0.19

Note: The comparisons were performed using Pearson's Chi‐square tests or Fisher's exact test, as appropriate, for categorical data, and Wilcoxon rank sum tests for continuous data. Countries included in the study were Cambodia, China, Hong Kong, India, Indonesia, Japan, Malaysia, Philippines, Singapore, South Korea, Taiwan, Thailand and Vietnam. Presented as *n* (%) for categorical data and median (interquartile range) for continuous data.

Abbreviations: ART, antiretroviral therapy; IDU, injecting drug use; NNRTI, non‐nucleoside reverse transcriptase inhibitor; PI, protease inhibitor; VL viral load.

Overall, the median frequency of VL measurement was 0.75 (0.15–1.24) time/year. For routine VL sites, it was 1.31 (1.17–2.00) time/year, and for non‐routine VL sites, it was 0.15 (0.10–0.66) time/year throughout the study period. The median duration on ART during the first‐line regimen for non‐routine VL sites was 9 (IQR 6–13) years with a total of follow‐up 29,671 PYs compared to 9 (IQR 5–13) years with a total of follow‐up 27,015 PYs for routine VL sites. For non‐routine VL sites, 849 (14%) patients were LTFU (2.86 per 100 PYs; 95% CI 2.68–3.06) and 76 (1%) died (mortality rate 0.26 per 100 PYs; 95% CI 0.20–0.32). The number of LTFU in routine VL was 524 (8%) with 1.94 per 100 PYs; (95% CI 1.78–2.11) and 136 (2%) died (mortality rate 0.50 per 100 PYs; 95% CI 0.43–0.60).

### 3.2 Virological failure

**Figure 2:**
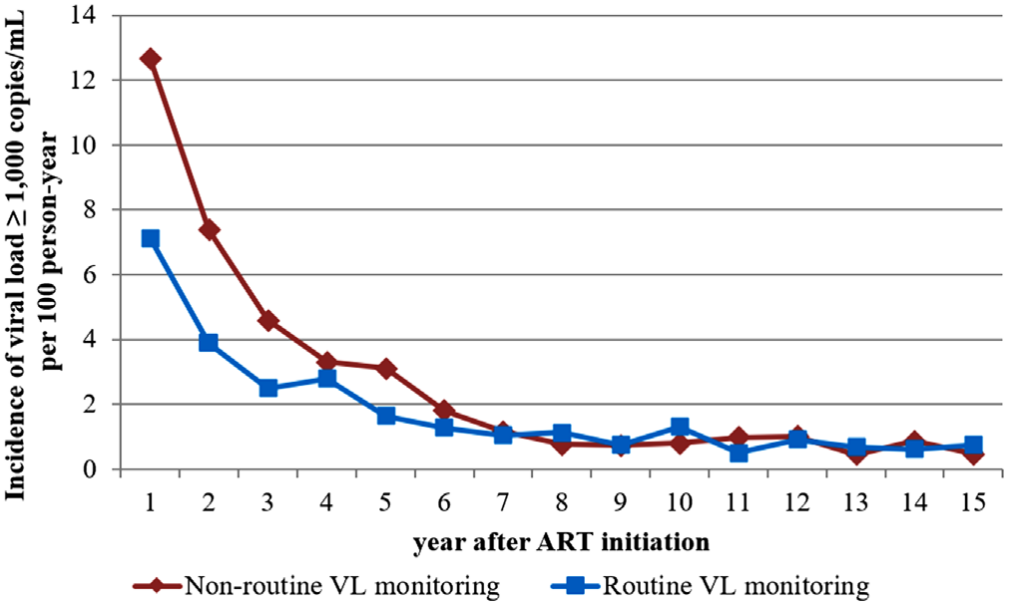
Incidence of viral load ≥1000 copies/ml after ART initiation during first‐line therapy.

Overall, 1021 subsequent VF at a rate of 2.15 (95% CI 2.02–2.29) per 100 PYs. We found that the proportion of VF on first‐line ART was 16% for non‐routine VL and routine VL sites. The crude VF rate of non‐routine VL (2.24 [95% CI 2.06–2.44] per 100 PYs) was higher than the crude rate of routine VL sites (2.06 [95% CI 1.89–2.26] per 100 PYs). Figure 2 shows differences between routine VL and non‐routine VL in VF rates in the first 3 years after ART initiation.

### 3.3 Factors associated with frequency of VF

Table 2 shows risk factors associated with VF on first‐line ART. We found that in the adjusted multivariate model, VF was more frequent at non‐routine VL sites (adjusted incidence rate ratio [aIRR] 2.85 [95% CI 2.27–3.59]) compared to routine VL sites. Patients with current age <25 years (aIRR 3.37, 95% CI 2.39–4.74), age 25 to <35 years (aIRR 2.74, 95% CI 2.28–3.30) and age 35 to <50 years (aIRR 1.79, 95% CI 1.52–2.12) were more likely to have VF when compared with those aged ≥50 years. The current CD4 count <350 cells/mm^3^ (aIRR 5.70, 95% CI 4.97–6.54) and unknown CD4 count (aIRR 3.33, 95% CI 2.86–3.87) were associated with a higher rate of VF when compared with those who had CD4 count ≥350 cells/mm^3^. Other factors associated with an increased rate of VF were HIV exposure and income country level.

**Table 2:** Factors associated with virological failure

Characteristics	Univariate		Multivariate	
	IRR (95% CI)	*p*	aIRR (95% CI)	*p*
VL monitoring status		<0.001		<0.001
Non‐routine VL	2.01 (1.78–2.27)		2.85 (2.27–3.59)	
Routine VL	reference		reference	
Income country level		<0.001		<0.001
Lower middle income	1.74 (1.46–2.07)		0.44 (0.32–0.60)	
Upper to middle income	1.10 (0.92–1.32)		0.67 (0.54–0.82)	
High income	reference		reference	
Sex		0.58		
Male	1.04 (0.91–1.18)			
Female	reference			
Current age, years		<0.001		<0.001
18 to <25	2.70 (1.97–3.72)		3.37 (2.39–4.74)	
25 to <35	2.58 (2.17–3.08)		2.74 (2.28–3.30)	
35 to <50	1.69 (1.44–1.98)		1.79 (1.52–2.12)	
≥ 50	reference		reference	
HIV exposure		<0.001		<0.001
Homosexual	reference		reference	
Heterosexual	1.37 (1.16–1.61)		1.32 (1.10–1.57)	
IDU	2.24 (1.51–3.32)		1.80 (1.22–2.66)	
Other	2.29 (1.85–2.83)		1.61 (1.30–2.00)	
Hepatitis C antibody status		<0.001		
Negative	reference		reference	0.80
Positive	1.61 (1.23–2.12)		0.96 (0.73–1.27)	
Unknown	1.57 (1.37–1.79)		1.54 (1.36–1.75)	
Prior AIDS diagnosis at ART initiation		<0.001		
Yes	reference		reference	0.07
No	1.36 (1.11–1.67)		1.20 (0.98–1.45)	
Unknown	0.85 (0.75–0.97)		0.85 (0.74–0.97)	
First major regimen		0.07		0.17
NNRTI‐based ART	1.22 (0.99–1.51)		0.94 (0.75–1.17)	
PI‐based ART	reference		reference	
Year of ART initiation		0.07		0.59
2003–2007	reference		reference	
2008–2013	0.88 (0.77–1.00)		0.89 (0.78–1.00)	
2014–2017	1.14 (0.88–1.46)		0.97 (0.75–1.26)	
2017–2021	1.12 (0.74–1.68)		0.87 (0.57–1.32)	
Current CD4 count, cells/mm^3^		<0.001		<0.001
<350	5.39 (4.74–6.13)		5.70 (4.97–6.54)	
≥350	reference		reference	
Unknown	3.54 (3.08–4.07)		3.33 (2.86–3.87)	

Abbreviations: aIRR, adjusted incidence rate ratio; ART, antiretroviral therapy; 95% CI, 95% confidence interval; IDU, injecting drug use; IRR, incidence rate ratio; NNRTI, non‐nucleoside reverse transcriptase inhibitor; PI, protease inhibitor; VL, viral load.

### 3.4 Switch to second‐line ART

A total of 817 (13%) patients switched to a second‐line ART regimen according to our definition. Of these, 481 (8%) patients switched due to VF, and 336 (5%) patients switched due to other reasons without evidence of VF. The median duration from VF to switching second‐line ART for PLHIV having VL results was 6 (2–23) months (7 [2–25] months in non‐routine VL sites and 5 [1–21] months in routine VL sites). In addition, the proportion of those who switched from routine VL sites was higher than non‐routine VL sites (18% vs. 9%). The overall rate of switching to second‐line ART was 1.44 (95% CI 1.35–1.54) per 100 PYs. The rate for routine VL sites was 1.97 (95% CI 1.81–2.15) per 100 PYs, which was higher than the rate for non‐routine VL sites (0.96 [95% CI 0.85–1.08] per 100 PYs, *p*‐value <0.001). The cumulative incidence of switching regimens in routine VL sites at 1, 2 and 3 years were 1.20% (95% CI 0.9–1.7%), 4.0% (95% CI 3.3–4.7%) and 6.2% (95% CI 5.4–7.1%), respectively. For non‐routine VL sites, the cumulative incidence of switching regimens at 1, 2 and 3 years were 0.2% (95% CI 0.1–0.5%), 1.3% (95% CI 1.0–1.8%) and 2.7% (95% CI 2.2–3.3%), respectively.

### 3.5 Factors associated with switching to second‐line ART

In the adjusted competing risk regression (Table 3), patients at sites with routine VL monitoring were at higher risk of switching compared with those at non‐routine VL sites (adjusted sub‐hazard ratio [aSHR] 1.78, 95% CI [1.17–2.71]). Switching to second‐line regimens was also more likely in high‐income countries (aSHR 1.79, 95% CI 1.09–2.96) compared to lower‐middle‐income countries. Switching regimen rates were higher in men (aSHR 1.23, 95% CI [1.02–1.48]) than in women. Patients aged<25 years (aSHR 11.38, 95% CI 5.63–23.00), age 25 to <35 years (aSHR 5.92, 95% CI 4.71–7.46) and age 35 to<50 years (aSHR 2.32, 95% CI 1.93–2.79) were more likely to switch to second‐line ART when compared with those aged ≥50 years. The time updated CD4 count <500 cells/mm^3^ (aSHR 3.32, 95% CI 2.84–3.89) was associated with a higher rate of switching regimen when compared with those who had CD4 count ≥500 cells/mm^3^. Other factors associated with increased risk of switching were HIV exposure, year of ART initiation and PI‐based regimen initiation.

**Table 3:** Factors associated with switching to second‐line regimen

Characteristics	Univariate		Multivariate	
	SHR (95% CI)	*p*	aSHR (95% CI)	*p*
VL monitoring status		<0.001		0.03
Non‐routine VL	reference		reference	
Routine VL	2.16 (1.87–2.49)		1.78 (1.17–2.71)	
Income country level		<0.001		<0.001
Lower middle income	reference		reference	
Upper to middle income	0.83 (0.69–1.00)		0.38 (0.25–0.58)	
High income	4.25 (3.62–4.99)		1.79 (1.09–2.96)	
Sex		<0.001		0.03
Male	1.63 (1.38–1.92)		1.23 (1.02–1.48)	
Female	reference		reference	
Current age, years		<0.001		<0.001
18 to <25	7.47 (4.41–12.64)		11.38 (5.63–23.00)	
25 to <35	4.85 (3.96–5.96)		5.92 (4.71–7.46)	
35 to <50	1.89 (1.58–2.26)		2.32 (1.93–2.79)	
≥ 50	reference		reference	
HIV exposure		<0.001		<0.001
Homosexual	1.78 (1.53–2.08)		0.53 (0.42–0.65)	
Heterosexual	reference		reference	
IDU	0.57 (0.30–1.11)		0.52 (0.26–1.02)	
Other	1.16 (0.92–1.45)		0.65 (0.51–0.84)	
Hepatitis C antibody status		0.04		0.27
Negative	1.54 (1.09–2.17)		1.30 (0.88–1.92)	
Positive	reference		reference	
Unknown	1.45 (1.03–2.04)		1.57 (1.07–2.31)	
Prior AIDS diagnosis at ART initiation		0.55		
Yes	1.08 (0.84–1.37)			
No	reference			
Unknown	0.86 (0.68–1.09)			
First major regimen		<0.001		<0.001
NNRTI‐based ART	reference		reference	
PI‐based ART	3.23 (2.75–3.8)		1.34 (1.06–1.68)	
Year of ART initiation		<0.001		<0.001
2003–2007	reference		reference	
2008–2013	0.90 (0.78–1.03)		0.95 (0.82–1.11)	
2014–2017	0.52 (0.36–0.76)		0.73 (0.49–1.09)	
2017–2021	0.14 (0.05–0.38)		0.31 (0.11–0.87)	
Current CD4 count, cells/mm^3^		<0.001		<0.001
<350	3.23 (2.76–3.79)		3.32 (2.84–3.89)	
≥350	reference		reference	
Unknown	0.20 (0.15–0.28)		0.28 (0.20–0.38)	

Abbreviations: ART, antiretroviral therapy; aSHR, adjusted sub‐distribution hazard ratio; 95% CI, 95% confidence interval; IDU, injecting drug use; NNRTI, non‐nucleoside reverse transcriptase inhibitor; PI, protease inhibitor; SHR, sub‐distribution hazard ratio; VL, viral load.

## 4 DISCUSSION

VL testing can be used as a tool to monitor HIV treatment responses and inform decisions for treatment switching. In our study, the overall crude rate of the first VF after ART initiation at non‐routine VL sites was slightly higher than at the routine sites. The rate of subsequent VF during first‐line ART was higher in PLHIV at non‐routine VL sites compared with PLHIV at routine VL sites. As expected, the rate of switching to second‐line in PLHIV at non‐routine VL sites was lower than PLHIV at routine VL sites due to our definition of treatment failure, including virological, immunological and clinical failure. The median overall VL testing frequency for the cohort was 0.75 (0.15–1.24) time/year. The VL testing frequency at routine VL sites was higher than at non‐routine VL sites. We did not utilize a second confirmatory VL testing to define VF in order to capture all VF that occurred in our cohort. This finding indicated immediately switching to second‐line ART after detecting VF for routine VL sites were better clinical management and HIV treatment. Compared to non‐routine VL sites, the rate of switching regimens at routine VL was about two times by 3 years of first‐line therapy higher in PLHIV at routine VL sites. Moreover, younger aged < 25 years were at higher risk of treatment failure and switching to second‐line ART compared to those aged ≥ 25 years old in our study who need to concern for long‐term treatment outcome in this age group.

The implementation of routine VL testing is still a major challenge for the Asia‐Pacific region since the WHO's recommendations in 2017 [2]. We found that there was slightly higher rate of first VF among those from routine VL testing sites. This is similar to prior studies in Vietnam [6], Uganda [17] and Thailand [18] where patients with routine monitoring had a higher proportion of VF, but no differences in treatment outcomes. A previous study in Southern Africa shows that the increased VL testing can result in higher rates of viral suppression; however, there was no evidence of a decrease in patients with detectable VL with long‐term follow‐up [19]. In this study, we focused on the frequency of subsequent VF rate between non‐routine VL and routine VL sites during long‐term follow‐up of first‐line therapy. PLHIV at non‐routine VL testing sites were at 52% higher persistent VF rate than PLHIV at routine VL testing sites after adjusting with other factors. Non‐routine VL testing increased the rate of subsequent VF during follow‐up due to a lack of timely follow‐up and under‐utilized VL testing in resource‐limited settings [20, 21, 22]. Moreover, non‐routine VL was also associated with a long duration of occurring viremia which can cause virologic failure [23, 24]. Our study was intended to better understand the effect of subsequent VF between non‐routine VL and routine VL testing sites in long‐term follow‐up.

We also found that PLHIV from routine VL sites had a higher chance of switching to second‐line ART compared to non‐routine VL sites. This suggests that routine VL sites were able to identify treatment failure and switch patients promptly to second‐line ART.

Prior studies have indicated that scaling up routine VL testing can lead to earlier detection of treatment failure, which allows for immediate switches of ART regimens [7]. In resource‐limited settings, switching of ART normally occurred after a single VF, and most patients were switched based on clinical failure without laboratory evidence of treatment failure [5, 10, 25]. When routine VL monitoring is not available, there could be a delay in ART switches resulting in decrease in CD4 cell count. PLHIV with lower CD4 count were at higher risk of VF as well as switching of ART, similar to previous findings from Asia [14] and sub‐Saharan Africa [7]. ART programmes in sub‐Saharan Africa, Asia and Latin America indicated that low pre‐ART CD4 counts were associated with switching of ART regimens in sites with and without routine VL testing. Moreover, adherence challenges, the high cost or unavailability of second‐line regimens may be the reasons for delayed switching in low/upper middle‐income countries [8, 26, 27]. The lack of routine VL testing has also contributed to delayed VF detection and first‐line ART switches [28]. Switching to DTG as per WHO recommendation has been implemented across several countries. Our findings indicate that a small proportion of PLHIV (*n* = 84) switched to DTG as a second‐line regimen during the early period of WHO's recommendation for DTG use. Most of these PLHIV were from upper middle‐ and high‐income countries. The majority of PLHIV in our study have been using NNRTI‐based ART. There is a recommendation to switch to DTG in combination with an optimized NRTIs backbone as the preferred second‐line regimen [11]. This is much easier for PLHIV to take than the previous PI‐based second‐line ART and may also encourage more routine VL testing to detect early treatment failure in long‐term follow‐up.

Our study has strengths and limitations. One of the strengths is to indicate the IRR of subsequent VF between non‐routine VL and routine VL testing sites after adjusting with other factors during first‐line therapy. There are several limitations to our study. First, we defined VF according to a single VL ≥1000 copies/ml without secondary confirmatory testing. This may overestimate our VF proportions. However, to accommodate sites that do not perform routine VL testing or sites that do not perform confirmatory testing, we believe our definition of VF allowed us to identify VF in our cohort settings appropriately. Second, we did not assess treatment failures associated with routine and non‐routine CD4 testing. This would provide an alternative tool to identify risks in treatment outcomes for sites without routine VL testing. Third, as TAHOD patients were selected to be enrolled based on the likelihood of remaining in care, our results are not generalizable to the wider PLHIV population. Lastly, only a small proportion of PLHIV in our study switched to DTG. We believe that there could be a delay in the application of WHO's recommended HIV treatment policies in lower/upper middle‐income countries due to the varying costs and standard of care [29, 30]. The use of DTG in first‐line regimens can increase rapid viral suppression, which may encourage sites to perform routine VL testing in order to detect early treatment failure.

## 5 CONCLUSIONS

In conclusion, PLHIV from non‐routine VL sites had a higher incidence of persistent VF and low switching regimen rate with delay detecting VF, reflecting possible under‐utilized VL testing within these sites. However, the expanding access to routine VL should be continued for HIV treatment monitoring and more common to consider switching ART earlier. Findings suggest that different VL monitoring strategies may have an impact on the time of treatment failure and switch to second‐line, as well as the development of drug resistance in long‐term treatment.

## COMPETING INTERESTS

The authors declare they have no competing interests.

## AUTHORS’ CONTRIBUTIONS

ST designed the study, drafted the initial manuscript, reviewed and critically revised and approved the final manuscript as submitted. AJ was responsible for data aggregation and data management. ST conducted the analysis. AJ and ML advised on the analysis, revised and approved the final manuscript. All authors critically reviewed the manuscript and approved the manuscript for submission.

### TAHOD study members

PS Ly*, V Khol, National Center for HIV/AIDS, Dermatology & STDs, Phnom Penh, Cambodia; FJ Zhang*, HX Zhao, N Han, Beijing Ditan Hospital, Capital Medical University, Beijing, China; MP Lee*, PCK Li, TS Kwong, TH Li, Queen Elizabeth Hospital, Hong Kong SAR; N Kumarasamy*, C Ezhilarasi, Chennai Antiviral Research and Treatment Clinical Research Site (CART CRS), VHS‐Infectious Diseases Medical Centre, VHS, Chennai, India; S Pujari*, K Joshi, S Gaikwad, A Chitalikar, Institute of Infectious Diseases, Pune, India; RT Borse*, V Mave, I Marbaniang, S Nimkar, BJ Government Medical College and Sassoon General Hospital, Pune, India; TP Merati*, IKA Somia, AAS Sawitri, F Yuliana, Faculty of Medicine Udayana University & Sanglah Hospital, Bali, Indonesia; E Yunihastuti*, A Widhani, S Maria, TH Karjadi, Faculty of Medicine Universitas Indonesia ‐ Dr. Cipto Mangunkusumo General Hospital, Jakarta, Indonesia; J Tanuma*, S Oka, T Nishijima, National Center for Global Health and Medicine, Tokyo, Japan; JY Choi*, Na S, JM Kim, Division of Infectious Diseases, Department of Internal Medicine, Yonsei University College of Medicine, Seoul, South Korea; YM Gani*, NB Rudi, Hospital Sungai Buloh, Sungai Buloh, Malaysia; I Azwa*, A Kamarulzaman, SF Syed Omar, S Ponnampalavanar, University Malaya Medical Centre, Kuala Lumpur, Malaysia; R Ditangco*, MK Pasayan, ML Mationg, Research Institute for Tropical Medicine, Muntinlupa City, Philippines; YJ Chan*, WW Ku, PC Wu, E Ke, Taipei Veterans General Hospital, Taipei, Taiwan; OT Ng*, PL Lim, LS Lee, T Yap, Tan Tock Seng Hospital, National Centre for Infectious Diseases, Singapore (note: OT Ng is also supported by the Singapore Ministry of Health's (MOH) National Medical Research Council (NMRC) Clinician Scientist Award (MOH‐000276). Any opinions, findings and conclusions or recommendations expressed in this material are those of the author(s) and do not reflect the views of MOH/NMRC; A Avihingsanon*, S Gatechompol, P Phanuphak, C Phadungphon, HIV‐NAT/Thai Red Cross AIDS Research Centre, Bangkok, Thailand; S Kiertiburanakul*, A Phuphuakrat, L Chumla, N Sanmeema, Faculty of Medicine Ramathibodi Hospital, Mahidol University, Bangkok, Thailand; R Chaiwarith*, T Sirisanthana, J Praparattanapan, K Nuket, Chiang Mai University ‐ Research Institute for Health Sciences, Chiang Mai, Thailand; S Khusuwan*, P Payoong, P Kantipong, P Kambua, Chiangrai Prachanukroh Hospital, Chiang Rai, Thailand; TN Pham*, KV Nguyen, DTH Nguyen, DT Nguyen, National Hospital for Tropical Diseases, Hanoi, Vietnam; CD Do*, AV Ngo, LT Nguyen, Bach Mai Hospital, Hanoi, Vietnam; AH Sohn*, JL Ross*, B Petersen, TREAT Asia, amfAR ‐ The Foundation for AIDS Research, Bangkok, Thailand; MG Law*, A Jiamsakul*, D Rupasinghe, The Kirby Institute, UNSW Sydney, NSW, Australia. * TAHOD Steering Committee member.

## FUNDING

The Kirby Institute is funded by the Australian Government Department of Health and Ageing, and is affiliated with the Faculty of Medicine, UNSW Sydney. International SciKU Branding (ISB) is funded by the Faculty of Science, Kasetsart University.

## DISCLAIMER

Data were presented in part at the 4th Asia Pacific AIDS & Co‐infections Conference, June 27–29, 2019, HongKong. The content of this publication is solely the responsibility of the authors and does not necessarily represent the official views of any of the governments or institutions mentioned above.

## Data Availability

The data that support the findings of this study are available from the corresponding author upon reasonable request. Individuals who would like to assess the data from the IeDEA Asia‐Pacific consortium for research purposes may need to submit a concept proposal in the detail at https://www.iedea.org/.
